# Vitamin B12 deficiency is associated with adverse lipid profile in Europeans and Indians with type 2 diabetes

**DOI:** 10.1186/s12933-014-0129-4

**Published:** 2014-09-26

**Authors:** Antonysunil Adaikalakoteswari, Ramamurthy Jayashri, Nithya Sukumar, Hema Venkataraman, Rajendra Pradeepa, Kuppan Gokulakrishnan, Ranjit Mohan Anjana, Philip G McTernan, Gyanendra Tripathi, Vinod Patel, Sudhesh Kumar, Viswanathan Mohan, Ponnusamy Saravanan

**Affiliations:** Warwick Medical School, University of Warwick, Coventry, UK; Department of Epidemiology & Diabetology, Madras Diabetes Research Foundation & Dr.Mohan’s Diabetes Specialities Centre, WHO Collaborating Centre for Non-communicable Diseases Prevention and Control & IDF Centre of Education, 4, Conran Smith Road, Gopalapuram, Chennai, 600 086 India; Academic department of Diabetes and Metabolism, George Eliot Hospital, Nuneaton, UK; WISDEM centre, University Hospital Coventry and Warwickshire, Coventry, UK

## Abstract

**Background:**

Metformin, a standard therapy in type 2 diabetes, reduces vitamin B12 levels. Studies linking low vitamin B12 levels and cardiovascular disease are equivocal and suggest improving B12 levels may help in primary prevention. The role of vitamin B12 deficiency on cardiovascular risk factors, especially in type 2 diabetes has not been explored. The aim of this study is to investigate whether vitamin B12 deficiency in type 2 diabetes patients is associated with cardiovascular risk factors in two different ethnic groups in UK and India.

**Methods:**

Type 2 diabetes patients from two secondary care diabetic centres (Europeans - UK and Indians - India) were studied. Serum vitamin B12, folate and biochemical parameters were measured.

**Results:**

The prevalence rates of vitamin B12 deficiency (<191 ng/L) were 27% and 12% in Europeans and Indians, respectively and higher in metformin treated type 2 diabetes patients. In linear regression analysis, after adjusting for all likely confounding factors, vitamin B12 independently associated with triglycerides in both the populations and cholesterol/HDL ratio in Indians. Logistic regression showed type 2 diabetes patients with vitamin B12 deficiency were at significantly higher odds of having coexisting coronary artery disease (CAD) in Europeans with similar but non-significant trend in Indians, after adjusting for all likely confounding factors.

**Conclusions:**

The prevalence of vitamin B12 deficiency is common in type 2 diabetes patients and is associated with adverse lipid parameters. Type 2 diabetes management guidelines should include the recommendation for regular testing for B12 levels, especially for those on metformin.

**Electronic supplementary material:**

The online version of this article (doi:10.1186/s12933-014-0129-4) contains supplementary material, which is available to authorized users.

## Introduction

Vitamin B12 is a key micronutrient responsible for DNA methylation and has various metabolic roles ranging from lipid metabolism to endothelial dysfunction [1]. Studies show association of low vitamin B12 with macro-vascular diseases such as myocardial infarction [2] and cerebral ischemia [3] as well as coronary artery disease (CAD) [4]. However, a systematic review of all published cohort studies was inconclusive [5]. B12 deficiency causes micro-vascular complications such as neuropathy [6] and can worsen the existing neuropathy due to other conditions such as diabetes [7].

Metformin therapy is now considered a standard first line therapy for type 2 diabetes (ADA, NICE, EASD guidelines) [8,9] and is commonly used. Metformin reduces the circulating B12 levels by about 25% [10-12]. One cross-sectional study of 203 type 2 diabetes patients reported the prevalence of B12 deficiency is 22% [13]. However, only 60% of patients with B12 deficiency have anaemia [14] and at milder forms patients with B12 deficiency are asymptomatic. This highlights the importance of regular screening but none of the above mentioned guidelines recommend measuring B12 levels regularly in type 2 diabetes, even when they are on metformin.

Indians have higher risk of metabolic disorders including type 2 diabetes and cardiovascular diseases (CVD) compared to Europeans [15,16] and these diseases also occur at younger age [17]. They also have higher homocysteine levels, which have been mainly attributed to low B12 levels [18]. Vegetarianism is thought to be cause of such high prevalence of B12 deficiency in this population. Whether high prevalence of B12 deficiency contributes to higher risk of CVD is not known [19].

The purpose of our study is (1) to assess the prevalence of vitamin B12 deficiency in type 2 diabetes patients and (2) its association with cardiovascular risk factors and micro- and macro-vascular diseases in two different ethnic groups in UK and India.

## Methods

### Study population

Cross-sectional data from two different secondary care diabetic centres were utilized for this study. **(1) UK participants:** 342 consecutive patients of European origin with type 2 diabetes, who had their vitamin B12 and folate levels checked in the George Eliot Hospital (GEH), Nuneaton, UK. **(2) Indian participants:** 321 type 2 diabetes patients of Indian origin had their vitamin B12 checked at the Dr Mohan’s Diabetes Specialties Centre were included for the analysis. Patients who were taking vitamin supplements and who were pregnant were excluded from the study. Detailed history, anthropometric and biochemical measures such as age, sex, type of diabetes, duration of diabetes, HbA_1_C, smoking status, medications, blood pressure, micro- and macro-vascular complications of diabetes, lipid profile, vitamin B12 and folate levels were collected from both the study population. Information on dietary intake (vegetarian/non-vegetarian) was not collected. These were routine anonymous clinical data extracted from records.

### Analytical determinations

Serum glucose, HbA_1_C, cholesterol, triglycerides, HDL cholesterol were determined by standard methodologies followed in the respective labs in both the study population. LDL cholesterol was calculated using Friedewald formula. Serum B12 and folate were determined by electrochemiluminescent immunoassay using a Roche Cobas immunoassay analyzer (Roche Diagnostics UK, Burgess Hill, UK). The reference values in both the laboratories were as follows: 191–663 ng/L for vitamin B12 and 2.5-18.7 ug/L for folate. Vitamin B12 and folate deficiencies were defined as levels below 191 ng/L [20] and 2.5 μg/L [21], respectively.

### Definition of comorbidity

The following definitions were used to diagnose the comorbidity. Retinopathy: Digital retinal photographs were graded by trained ophthalmologists (India) or retinal graders (UK) by the ETDRS grading system. Neuropathy: Vibratory perception threshold of the great toe > mean + 2SD of healthy non-diabetic study population aged 20–45 years (cut point ≥20 V). Nephropathy: Albumin excretion ≥30 μg/mg of creatinine in urine sample after an overnight fast (microalbuminuria - 30–299 μg/mg of creatinine and macroalbuminuria - ≥300 μg/mg of creatinine). Patients with documented retinopathy, peripheral and autonomic neuropathy, and nephropathy were recorded individually and classified to have microvascular complications. Coronary artery disease (CAD): Past history of documented myocardial infarction, stable and unstable angina, coronary artery bypass graft, stent and/or electrocardiographic changes suggestive of ST segment depression and/or Q-wave changes using appropriate Minnesota codes. Cerebrovascular accidents (CVA): Past history of documented stroke (computed tomography, magnetic resonance imaging, or cerebral angiography). Peripheral vascular disease (PVD): Lack of peripheral pulses or Doppler studies with Ankle Brachial Index <0.9. Those with documented CAD, CVA and PVD were recorded individually and classified to have macrovascular complications.

### Statistical analysis

Continuous variables are reported as mean ± standard deviation (SD). Categorical variables are reported in percentages. The distributions of the parameters such as cholesterol, triglycerides, HDL, LDL, vitamin B12 concentrations were skewed; these data were log-transformed. Means of continuous variables were compared using independent t-tests. Bivariate correlations between different variables were done using Pearson correlation test. Risk variables that had significant association were included as independent variables in multiple linear regression analysis. Logistic regression analysis was used to examine the relation between vitamin B12 levels and the risk of micro- and macro-vascular complications. Associations between vitamin B12 and cardiovascular outcomes were adjusted for age, gender, BMI, duration of diabetes, smoking, HbA_1_C, cholesterol, HDL, triglycerides, systolic and diastolic pressure, use of metformin, statin and aspirin. *p* values of <0.05 were considered as statistically significant. All analyses were performed using IBM SPSS Statistics version 19 (IBM Corp, NY, USA).

## Results

The clinical characteristics of the study population are shown in Table 1. The use of metformin in Europeans was 65% and in Indians is 75%. The prevalence rates of serum vitamin B12 deficiency (<191 ng/L) in Europeans were 27% and Indians were 12% (Table 1). For those on metformin, these rates were 32.1 and 12.4%, respectively. There were no gender differences in vitamin B12 and folic acid (Additional file 1: Table S1). The sex specific values of the other variables are also shown in the Additional file 1: Table S1. In both the populations, triglycerides and cholesterol/HDL ratio were significantly and inversely associated with vitamin B12 levels. HDL was positively associated with vitamin B12 levels in Europeans but cholesterol was not significantly associated with vitamin B12 in both the populations (Additional file 1: Table S2). No associations of vitamin B12 with other established cardiovascular risk factors such as BMI, systolic and diastolic pressure and HbA_1_C were observed (data not shown).

**Table 1:** **Basic Characteristics of the study population**

**Parameters**	**Europeans**	**Indians**
	**total**	**total**
	**n = 342**	**n = 321**
Age (years)	63.0 ± 12.3^a^	56.8 ± 10.6
BMI (Kg/m2)	32.8 ± 6.1	28.0 ± 5.7
Duration of diabetes (years)	14.1 ± 9.4	8.4 ± 7.6
HbA1C (%)	7.89 ± 1.62	8.30 ± 2.1
Cholesterol (mmol/L)	4.10 ± 1.10	4.0 ± 1.12
Triglycerides (mmol/L)	2.01 ± 1.48	1.77 ± 0.89
HDL (mmol/L)	1.25 ± 0.35	0.98 ± 0.25
LDL (mmol/L)	1.97 ± 0.81	2.20 ± 0.91
Cholesterol/HDL ratio	3.46 ± 1.19	4.18 ± 1.16
SBP (mmHg)	137 ± 20	132 ± 18
DBP (mmHg)	74 ± 11	81 ± 9.3
Vitamin B12 (ng/L)	290 ± 139	464 ± 228
Vitamin B12 deficiency, n (%)	91 (27)^b^	37 (12)
Folate (ug/L)	7.71 ± 9.77	13.6 ± 5.2
Folate deficiency, n (%)	29 (8.5)	0
Smoking, n (%)	26 (8.5)	75 (24)
Microvascular complications:		
Retinopathy, n (%)	124 (36)	132 (45)
Neuropathy, n (%)	53 (16)	99 (33)
Nephropathy, n (%)	41 (12)	83 (29)
Macrovasuclar complications:		
Coronary artery disease (CAD), n (%)	62 (18)	27 (9)
Cerebro vascular accidents (CVA), n (%)	19 (5.6)	6 (1.9)
Peripheral vascular disease (PVD), n (%)	23 (6.7)	17 (6)
Insulin use, n (%)	215 (63)	149 (46)
Metformin use, n (%)	221 (65)	242 (75)
Statin use, n (%)	286 (84)	154 (48)
Aspirin use, n (%)	246 (72)	44 (14)

^a^Mean ± SD (all such values); ^b^Numbers (percentages) (all such values).

**Figure 1:**
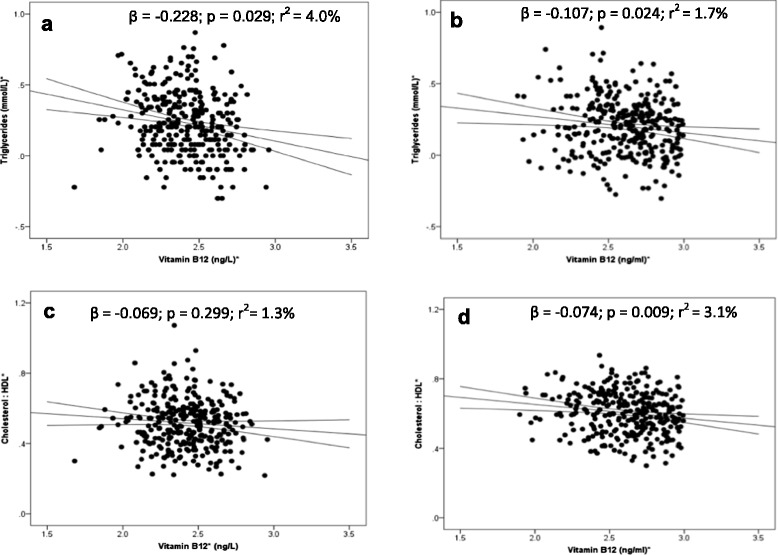
**Regression of Vitamin B12 with (a) Triglycerides in Europeans, (b) Triglycerides in Indians, (c) Cholesterol: HDL ratio in Europeans and (d) Cholesterol: HDL ratio in Indians.** *Log-transformed for statistical comparisons. Model included vitamin B12, age, BMI, sex, duration of diabetes, HbA_1_C, smoking, use of metformin, statin & aspirin.

Linear regression analysis was carried out to assess whether vitamin B12 independently associated with these cardiovascular risk factors in the type 2 diabetes patients by adjusting for all likely confounders. The model included age, gender, BMI, duration of diabetes, smoking, HbA_1_C, use of metformin, statin and aspirin as independent variables. After all these adjustments, vitamin B12 independently associated with triglycerides in both the populations (Figure 1a,b) but cholesterol/HDL ratio only in Indians (Figure 1d).

Association of vitamin B12 levels and individual comorbidities were assessed by logistic regression analysis (Table 2). After adjustment for age, gender, BMI, duration of diabetes, smoking, HbA_1_C, cholesterol, HDL, LDL, triglycerides, systolic and diastolic pressure, use of metformin, statin and aspirin, type 2 diabetes patients with low vitamin B12 levels were at a significantly higher odds of having coexisting CAD in Europeans (OR = 3.91; 95% CI: 1.09 - 14.05). A similar but non-significant trend of higher risk was observed in Indians (OR = 1.77; 95% CI: 0.376 - 8.33). No associations with other micro- or macro- vascular diseases were observed.

**Table 2:** **Logistic regression analysis of vitamin B12 with co-morbidities**

**Co-morbidities**	**Europeans**			**Indians**		
	**B (SE)**	**Odds ratio (95% CI)**	**p-value**	**B (SE)**	**Odds ratio (95% CI)**	**p-value**
**Microvascular complications:**						
Retinopathy	0.294 (0.604)	1.342 (0.411, 4.386)	0.626	−0.085 (0.440)	0.919 (0.388, 2.176)	0.848
Neuropathy	0.132 (0.663)	1.141 (0.311, 4.186)	0.842	−0.208 (0.558)	0.812 (0.272, 2.424)	0.709
Nephropathy	0.790 (1.132)	2.203 (0.239, 20.267)	0.485	−0.721 (0.519)	0.487 (0.176, 1.346)	0.165
**Macrovascular complications:**						
Coronary artery disease (CAD)	1.364 (0.653)	3.911 (1.088, 14.054)	0.037	0.571 (0.790)	1.770 (0.376, 8.332)	0.470
Cerebro vascular accidents (CVA)	−1.226 (1.508)	0.294 (0.015, 5.644)	0.416	0.775 (1.584)	2.170 (0.097, 48.369)	0.625
Peripheral vascular disease (PVD)	0.552 (0.608)	1.737 (0.527, 5.722)	0.364	-	-*	-

Model included vitamin B12, age, BMI, sex, duration of diabetes, HbA_1_C, Cholesterol, HDL, LDL, triglycerides, SBP, DBP, smoking, use of metformin, statin and aspirin.

*- Odds ratio cannot be computed because in the PVD group, the number of B12 deficient cases were zero.

## Discussion

Our study involving two different ethnic groups with type 2 diabetes patients had three main findings. Firstly, there was high prevalence of vitamin B12 deficiency in Europeans but interestingly lower than observed prevalence in Indians from South India. Secondly, vitamin B12 deficiency was associated with adverse lipid profiles. Thirdly, low vitamin B12 levels in type 2 diabetes patients were associated with an increased risk of CAD.

Studies in type 2 diabetes patients of European origin on metformin have reported the prevalence of vitamin B12 deficiency to range from 5.8% to 33% [10,11,13,22]. Our study confirms this in the British population, with a prevalence of 27% in all type 2 diabetes and 32.1% in type 2 diabetes with metformin. Our study is the first one to report the prevalence of B12 deficiency in the South Indian population with type 2 diabetes. Previous studies in north Indian population showed much higher rates of 67% in middle-aged men [18] and 54% in diabetes patients [23]. This is likely due to the differences in dietary habits between north and south Indians. South Indians consume higher quantity of fermented foods, which are rich in vitamin B12 [24,25].

In this study, vitamin B12 deficiency independently associated with triglycerides and cholesterol/HDL ratio in type 2 diabetes patients. Our findings were similar to the study in Indians with history of CAD [4]. Similar correlations were also found between B12 levels and total cholesterol and triglycerides in a group of Polish patients with established atherosclerosis, but this relationship was lost in regression analysis, which may be due to the smaller sample size in the study [21]. Vitamin B12 functions as a coenzyme in the conversion of methylmalonyl-CoA (MM-CoA) to succinyl-CoA [26,27]. This reaction is blocked if there is vitamin B12 deficiency, resulting in accumulation of MM-CoA which inhibits the rate-limiting enzyme of fatty acid oxidation (CPT1 – carnitine palmitoyl transferase) [28], thus causing lipogenesis. This may be the likely mechanism for the link between B12 deficiency and adverse lipid parameters.

Our observation of an association of increased risk of CAD in type 2 diabetes patients with low B12 levels after controlling for all likely confounding factors is supported by other findings in subjects with type 2 diabetes and non-type 2 diabetes. A study by Shargorodsky *et al.* found that vitamin B12 independently correlated with pulse wave velocity in type 2 diabetes patients, an accepted cardiovascular risk factor [29]. Weikert *et al.* [3] in a population-based prospective study showed the association between low vitamin B12 levels and increased risk of cerebral ischemia. Similarly, in south Asian women living in the UK with vitamin B12 deficiency anaemia had a higher prevalence of myocardial infarction and CAD [30]. We did not find any sex specific changes in our study. Thus our findings in support of the previous observations extend the knowledge on the role of vitamin B12 on CAD and its risk factors in type 2 diabetes patients. In spite of the fact that B-vitamins could provide an inexpensive and effective method for the prevention of CVD, their use was rejected, based on the negative results of randomized controlled clinical trials [31,32]. But, when examining the design of these trials, it appeared that concomitant medication such as statin/aspirin therapy applied along with the vitamin substitution could have obscured the separate effects of vitamins in cardiovascular prevention. However, a recent meta-analysis of these vitamin trials suggest that B vitamins are effective in primary prevention of cardiovascular diseases [33]. Similarly, a study in type 2 diabetes patients with another micronutrient supplementation, vitamin D, showed more significant improvements in the cardio-metabolic profile [34].

Lipid abnormalities are unique in individuals with T2D and those are at risk of T2D (obesity, metabolic syndrome and pre-diabetes): the total cholesterol and LDL are lower in those with statins but higher in those without. In addition, in both groups the triglyceride levels are higher and the HDL levels are lower as statins have little effect on them [35-37]. In post-menopausal women with T2D and CAD who were not on lipid lowering medications, in addition to higher total and LDL cholesterol and higher triglycerides, homocysteine was also higher, suggesting a potential link between vitamin B12 and folic acid and abnormal lipid profiles [38-41]. It is known that increasing triglycerides and reducing HDL are early features of atherosclerosis, well before increasing LDL [38,42]. Therefore, our findings showing independent association of B12 with triglycerides and HDL in two different ethnic groups provide a possible mechanism how vitamin B12 could offer primary prevention of cardiovascular diseases in type 2 diabetes and may also be an option in the secondary prevention of disease, if statin therapy is accompanied by serious adverse effects.

The strength of this study is the inclusion of two cross-sectional study populations of type 2 diabetes patients from UK and India and comprehensive data from both the groups. However, it also has the following limitations. The study population were based in secondary care and not a true representative sample of all type 2 diabetes. A true primary care representative sampling of type 2 diabetes would have strengthened our findings. However, metformin is routinely prescribed in primary care. This therefore might have under estimated the prevalence of B12 deficiency and may have overestimated its association with CAD as the prevalence of CAD is likely to be higher in secondary care settings. A group of Indian diaspora living in the UK as well as the availability of biomarkers of vitamin B12 deficiency such as MMA and homocysteine would have strengthened the findings. In addition, being a cross-sectional study, it does not prove causality.

In summary, our study demonstrates for the first time that vitamin B12 deficiency in type 2 diabetes patients in two different ethnic groups is associated with adverse lipid parameters and higher risk of CAD. Currently, there are no guidelines advocating for routine screening for vitamin B12 deficiency among patients with type 2 diabetes. While optimal screening frequency remains to be determined, baseline tests at initiation of metformin therapy and subsequent annual testing of B12 levels may be appropriate. Our study also warrants updating of international guidelines for the management of type 2 diabetes.

### Statement of human rights

This study was conducted with routine health care data. Therefore, full protocol review was not required. “Ethical approval: For this type of study formal consent is not required”.

### Informed consent

Informed consent from all patients in the study was not necessary.

## Additional file

Additional file 1: Table S1. Basic Characteristics of the study population. **Table S2.** Correlation between Vitamin B12 and lipid profile.
