# Supplementary material for: Vitamin B12 deficiency is associated with adverse lipid profile in Europeans and Indians with type 2 diabetes
Source: Cardiovasc Diabetol. 2014 Sep 26;13:129. doi: 10.1186/s12933-014-0129-4 (PMC4189588; doi:10.1186/s12933-014-0129-4)
Supplement: Additional file 1: Table S1. — Basic Characteristics of the study population. Table S2. Correlation between Vitamin B12 and lipid profile. [file 12933_2014_129_MOESM1_ESM.docx]

**Additional file 1:** **Table S1. Basic Characteristics of the study population:**

| **Parameters** | **Europeans** | | |  | **Indians** | | |
| --- | --- | --- | --- | --- | --- | --- | --- |
|  | **Total subjects** | **Male** | **Female** |  | **Total subjects** | **Male** | **Female** |
|  | n=342 | n=180 | n=162 |  | n=321 | n=201 | n=120 |
| Age (years) | 63.0 ± 12.3^a^ | 62.4 ± 11.2 | 63.7 ± 13.4 |  | 56.8 ± 10.6 | 57.1 ±11.3 | 56.26 ± 9.4 |
| BMI (Kg/m2) | 32.8 ± 6.1 | 32.3 ± 6.3 | 33.5 ± 5.8 |  | 28.0 ± 5.7 | 27.3 ± 4.5 | 29 ± 7.1 |
| Duration of Diabetes (years) | 14.1 ± 9.4 | 14.2 ± 9.9 | 14.0 ± 8.9 |  | 8.4 ± 7.6 | 9.2 ± 7.9 | 7.1 ± 6.8 |
| HbA1C (%) | 7.89 ± 1.62 | 7.83 ± 1.50 | 7.96 ± 1.74 |  | 8.3 ± 2.1 | 8.3 ± 2.1 | 8.4 ± 2.1 |
| Cholesterol (mmol/L) | 4.10 ± 1.10 | 3.93 ± 1.17 | 4.29 ± 1.00 |  | 4.00 ± 1.12 | 3.93 ± 1.18 | 4.11 ± 0.99 |
| Triglycerides (mmol/L) | 2.01 ± 1.48 | 2.09 ± 1.77 | 1.92 ± 1.07 |  | 1.77± 0.89 | 1.81 ± 0.95 | 1.69 ± 0.75 |
| HDL (mmol/L) | 1.25 ± 0.35 | 1.14 ± 0.28 | 1.37 ± 0.37 |  | 0.98 ± 0.25 | 0.94 ± 0.22 | 1.05 ± 0.28 |
| LDL (mmol/L) | 1.97 ± 0.81 | 1.86 ± 0.80 | 2.08 ± 0.81 |  | 2.20 ± 0.91 | 2.16 ± 0.97 | 2.26 ± 0.81 |
| Cholesterol/HDL ratio | 3.46 ± 1.19 | 3.60 ± 1.28 | 3.32 ± 1.09 |  | 4.18 ± 1.16 | 4.28 ± 1.18 | 4.01 ± 1.10 |
| SBP (mmHg) | 137 ± 20 | 137 ± 19 | 137 ± 21 |  | 132 ± 18 | 131 ± 18 | 133 ± 19 |
| DBP (mmHg) | 74 ± 11 | 75 ± 11 | 72 ± 11 |  | 81 ± 9.3 | 81 ± 9.2 | 81 ± 9.6 |
| Vitamin B12 (ng/L) | 290 ± 139 | 276 ± 136 | 305 ± 146 |  | 464 ± 228 | 448 ± 231 | 491 ± 220 |
| Vitamin B12 deficiency, n (%) | 91 (27)^b^ | 52 (29) | 39 (24) |  | 37 (12) | 28 (14) | 9 (8) |
| Folate (ug/L) | 7.71 ± 9.77 | 8.27 ± 12.7 | 7.09 ± 4.76 |  | 13.6 ± 5.2 | 13.3 ± 5.2 | 14.2 ± 5.4 |
| Folate deficiency, n (%) | 29 (8.5) | 15 (8.4) | 14 (8.6) |  | 0 | 0 | 0 |
| Smoking, n (%) | 26 (8.5) | 14 (8.6) | 12 (8.3) |  | 75 (24) | 74 (37) | 1 (0.8) |
| Microvascular complications: |  |  |  |  |  |  |  |
| Retinopathy, n (%) | 124 (36) | 71 (39) | 53 (33) |  | 132 (45) | 88 (47) | 44 (40) |
| Neuropathy, n (%) | 53 (16) | 35 (19) | 18 (11) |  | 99 (33) | 63 (34) | 36 (32) |
| Nephropathy, n (%) | 41 (12) | 25 (14) | 16 (10) |  | 83 (29) | 58 (34) | 25 (22) |
| Macrovasuclar complications: |  |  |  |  |  |  |  |
| Coronary artery disease (CAD), n (%) | 62 (18) | 37 (21) | 25 (15) |  | 27 (9) | 21 (11) | 6 (5) |
| Cerebro vascular accidents (CVA), n (%) | 19 (5.6) | 11 (6.1) | 8 (4.9) |  | 6 (1.9) | 5 (2.5) | 1 (0.8) |
| Peripheral vascular disease (PVD), n (%) | 23 (6.7) | 17 (9.4) | 6 (3.7) |  | 17 (6) | 11 (7) | 6 (5) |
| Insulin use, n (%) | 215 (63) | 115 (64) | 100 (62) |  | 149 (46) | 98 (49) | 51 (43) |
| Metformin use, n (%) | 221 (65) | 116 (64) | 105 (65) |  | 242 (75) | 145 (72) | 97 (81) |
| Statin use, n (%) | 286 (84) | 154 (86) | 132 (82) |  | 154 (48) | 100 (50) | 54 (45) |
| Aspirin use, n (%) | 246 (72) | 134 (74) | 112 (69) |  | 44 (14) | 33 (16) | 11 (9) |

^a^Mean ± SD (all such values) ; ^b^Numbers (percentages) (all such values)

**Additional file 1:** **Table S2. Correlation between Vitamin B12 and lipid profile**

| **Parameters** | **Europeans** | | | | | |  | **Indians** | | | | | |
| --- | --- | --- | --- | --- | --- | --- | --- | --- | --- | --- | --- | --- | --- |
|  | **Total subjects**  n=342 | | **Males**  n=180 | | **Females**  n=162 | |  | **Total subjects**  n=321 | | **Males**  n=201 | | **Females**  n=120 | |
|  | Vitamin B12 | | | | | |  | Vitamin B12 | | | | | |
|  | r-value | p-value | r-value | p-value | r-value | p-value |  | r-value | p-value | r-value | p-value | r-value | p-value |
| Cholesterol | 0.011 | 0.840 | -0.038 | 0.618 | 0.072 | 0.370 |  | -0.089 | 0.112 | -0.150 | 0.033 | 0.015 | 0.866 |
| Triglycerides | -0.193 | 0.001 | -0.185 | 0.019 | -0.198 | 0.015 |  | -0.133 | 0.018 | -0.219 | 0.002 | 0.033 | 0.713 |
| HDL | 0.179 | 0.002 | 0.177 | 0.024 | 0.106 | 0.200 |  | 0.063 | 0.259 | 0.054 | 0.440 | 0.031 | 0.740 |
| LDL | 0.075 | 0.198 | 0.051 | 0.535 | 0.130 | 0.120 |  | -0.065 | 0.245 | -0.099 | 0.159 | -0.009 | 0.921 |
| Cholesterol/HDL ratio | -0.110 | 0.052 | -0.135 | 0.087 | -0.150 | 0.854 |  | -0.114 | 0.041 | -0.218 | 0.002 | 0.026 | 0.797 |
